# Treatment of Recurrent Painful Ophthalmoplegic Neuropathy: A Case Where Pregabalin Was Successfully Employed

**DOI:** 10.1155/2019/9185603

**Published:** 2019-02-07

**Authors:** Laura N. Zamproni, Reinaldo T. Ribeiro, Marina Cardeal

**Affiliations:** Instituto de Neurologia Funcional, Rua Padre Machado, 42 São Paulo, SP, Brazil

## Abstract

Recurrent painful ophthalmoplegic neuropathy, previously known as ophthalmoplegic migraine, is a rare condition that affects children and young adults. Its cause and classification are still controversial and, consequently, there are no published treatment guidelines or consensus. Glucocorticoids seem to be beneficial for some patients, but there is no established treatment when failure of this therapy occurs. The aim of this study was to report a case where pregabalin was successfully used after failure of glucocorticoid therapy in a patient with recurrent painful ophthalmoplegic neuropathy.

## 1. Introduction

Recurrent painful ophthalmoplegic neuropathy (RPON), previously known as ophthalmoplegic migraine (OM), is a rare condition (estimated annual incidence of 0,7 per million) involving headache and ophthalmoplegia, which typically occurs in children and young adults [1]. Typical features include repeated attacks of paralysis of one or more ocular cranial nerves (III, IV, or VI) with ipsilateral headache that can start up to two weeks before the onset of eye muscle weakness [15]. The International Classification of Headache Disorders 3rd edition-beta version (ICHD3*β*) defined the following diagnostic criteria for RPON: (1) at least two attacks; (2) unilateral headache accompanied by ipsilateral paresis of one, two, or three ocular motor nerves; (3) the exclusion of orbital, parasellar, or posterior fossa lesions by an appropriate investigation; and (4) the absence of another diagnosis that could better account for the patient's condition [16].

RPON was used to be classified as a migraine variant, but the disease concept has changed due to lack of migraine symptoms in many cases and evidence supporting neuropathy [1]. Brain MRI reveals gadolinium enhancement of the affected cranial nerve in approximately 75 % of patients suggesting that the condition is a demyelinating disorder [15]. However, the cause and classification remain controversial.

This lack of information about the disease pathophysiology makes the treatment even more controversial. In fact, there are very few data or consensus about RPON treatment. The aim of this study is to report a case where pregabalin was successfully employed after glucocorticoid therapy failure in RPON.

## 2. Case Report

A 21-year-old female patient came to our service with a complaint of unilateral right-onset headache associated with diplopia initiated 6 months earlier. She had no personal or family remarkable antecedents. She never smoked. Six months earlier, the patient started to experience one-sided right throbbing headache. She denied nausea, vomiting, or photo- or phonophobia. Fifteen days after the pain onset, she noticed double vision and medial deviation of the right eye, which forced her to wear an eyepiece to perform her activities and drive. She went to several centers and used various medications such as paracetamol, nonsteroidal anti-inflammatory drugs (NSAIDs), opioids, and triptans without improvement. Three months earlier, she started using dexamethasone 4 mg daily with partial pain control but maintenance of diplopia.

**Figure 1:**
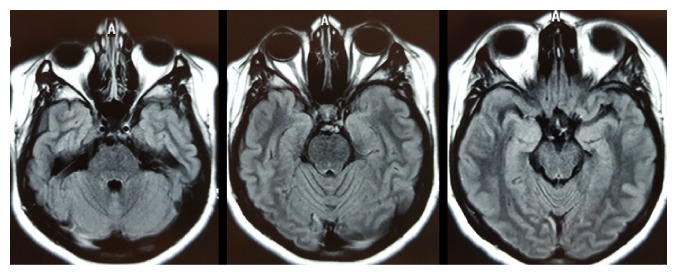
Axial flair brain MRI.

At the examination, the patient had cushingoid face, violaceous striae, and right VI cranial nerve palsy with no other neurological changes. Blood tests were normal (Table 1). A contrast-enhanced MRI scan of the brain did not show any remarkable features (Figure 1). A spinal tap released crystalline cerebrospinal fluid (CSF) with an opening pressure of 14 cm of water. Biochemical, microbiological, and cytological analyses of the CSF were normal (Table 1). CT scan of thorax did not show any evidence of lymphoma or sarcoidosis.

Prednisone 1mg / kg was then started. With one week of treatment, complete reversal of ocular paralysis and remission of pain were observed. However, when the corticoid was gradually withdrawn, the patient returned to pain and returned to paralysis of the VI right pair. The prednisone was increased again to 1 mg / kg this time with reversion of ocular paralysis but without pain control. Several prophylaxis attempts were made with beta-blockers, calcium channel blockers, topiramate, and tricyclics without any symptomatic control that would allow corticosteroid withdrawal.

The pregabalin 150mg daily was then introduced. With 7 days of medication onset there was already an important remission of pain. With 15 days of pregabalin initiation, the retitration of prednisone was started without any intercurrence and the patient reversed the exogenous Cushing syndrome. Pregabalin was maintained for one year and retracted. Currently, the patient has been free of pain for 2 years.

## 3. Discussion

According to the prevailing diagnostic criteria, our patients suffered from RPON given the presence of a documented VI nerve palsy associated with a unilateral ipsilateral headache with no remarkable alterations in laboratory or neuroimaging exams. Although the enhancement of the affected nerve occurs in 75% of patients [15], brain MRI is normal in a quarter of cases. We emphasize that our patient had been using a lower dose of corticosteroids for almost 3 months before admission, which may have reduced the sensitivity of the exam (Figure 1). Our patient responded partially to corticosteroids and absolutely to pregabalin but not to other treatment.

Since RPON is a very rare condition, it is difficult to conduct studies. There are no published treatment trials or guidelines for RPON and all evidence of effective treatments came from observational studies. Glucocorticoids seem to be beneficial for some patients. Prompt administration of steroids at the clinical onset may minimize permanent ophthalmoparesis or pupillary disfunction [17]. In a retrospective review of 80 patients, steroids were used to treat at least 31% of patients and there was a perceived benefit from corticosteroid treatment in 54 %. Only 12 % had no improvement or clinical worsening [15].

For those patients that do not respond to corticosteroids, treatment evidence is even smaller. The efficacy of drugs for migraine such as acetaminophen, NSAIDs, ergotamine, and triptans is unknown [1]. Pareja et al. described two cases of PRON responsive to indomethacin [18]. Also, cyproheptadine hydrochloride has been reported to prevent recurrent attacks of RPON by targeting the vascular edema around the oculomotor nerve [1].

Some authors recommend standard migraine prophylactic therapy for patients with recurrent attacks. We searched PubMed for case reports published between 2103 and 2017 using the search terms “ophthalmoplegic migraine” and “recurrent painful ophthalmoplegic neuropathy”. We found 10 case reports that are displayed at Table 2. After steroids, valproate and topiramate were the most prescribed medication. Both valproate and topiramate increase gamma-aminobutyric acid (GABA) levels in the brain and are first-line agents in migraine prevention (Table 3) [13].

In more refractory cases, injection of botulinum toxin or strabismus surgery may be considered for patients with persistent sequelae, to correct eye misalignment [15].

As far as we know, pregabalin has not been reported for RPON treatment. Pregabalin is a structural analogue of GABA. Pregabalin binds to alpha 2-delta subunits of the voltage-gated calcium cannels in the nervous system, reducing the influx of calcium in the presynaptic ending, and, consequently, reduces the release of excitatory neurotransmitters including glutamate, noradrenaline, dopamine, and serotonin in the synaptic cleft [14]. Pregabalin has been used to treat neuropathic pain. The response to pregabalin and the lack of efficacy of classic drugs used in migraine, such as NSAIDs and first-line prophylactic migraine drugs (topiramate and valproate) in this case, reinforce the neuropathic etiology of the disease and not a variant of migraine as it was formerly classified.

## Figures and Tables

**Table 1:** Results of blood and cerebrospinal fluid tests.

Hemoglobin (g/dL)	13.1

WBC (/mm^3^)	12700

Platelets (/mm^3^)	298000

Na (mmol/L)	141

K (mmol/L)	4,1

Coagulation tests	Normal

Creatinine (mg/dL)	0,5

CPK (U/L)	38

ESR (mm/h)	22

Bilirubin (mg/dL)	0,27

AST (U/L)	16

ALT (U/L)	18

ALP(U/L)	64

GGT (U/L)	47

TSH (um/L)	0,8

ANA	Negative

Anti-dsDNA	Negative

Anti-RNP	Negative

Anti-LA	Negative

Anti-RO	Negative

Anti-SM	Negative

ANCA	Negative

HIV	Negative

ACE (U/L)	31

VDRL test	Negative

Cerebrospinal fluid	

WBC (/mm^3^)	1

Protein (mg/dL)	22

Glucose (mg/dL)	66

Bacteria	Negative

WBC: white blood cells; Na: sodium; K: potassium; ESR: erythrocyte sedimentation rate; CPK: creatine phosphokinase; AST: aspartate aminotransferase; ALT: alanine transaminase; ALP: *alkaline phosphatase;  GGT:* gamma glutamyl transferase; TSH: thyroid stimulating hormone; ANA: antinuclear antibodies; Anti-dsDNA: anti-double stranded DNA antibodies; HIV: human immunodeficiency virus; ACE: angiotensin-converting enzyme; ANCA: anti-neutrophil cytoplasmic antibody; VDRL test: Venereal Disease Research Laboratory test.

**Table 2:** Case reports.

Author	Patient age/ gender	Pain description	Nerve palsy	Treatment	Time to fully recovery
Kobayashi et al. 2017 [1]	48-year-old /female	Unilateral Throbbing Photophobia Phonophobia	Incomplete III pair	Corticosteroids	5 days Persistent Residual Mydriasis

Qureshi et al. 2017 [2]	53-year-old / female	Unilateral Throbbing Photophobia Phonophobia	Incomplete III pair	NSAIDs Serotonin receptor agonist Calcium channel blocker	NR

Huang et al. 2017 [3]	26-year-old / female	Unilateral	Incomplete III pair	Corticosteroids Topiramate	6 weeks

Tripathi et al. 2017 [4]	36-year old / female	Unilateral Throbbing Photophobia	VI pair	Gabapentin Topiramate	2 months

Okura et al. 2017 [5]	12-year-old / male	Unilateral	Incomplete III pair	Corticosteroids	8 weeks

Takizawa et al. 2016 [6]	42-year-old / female	Unilateral Throbbing	Incomplete III pair	NR	NR

Ghosh 2015 [7]	5-year-old / male	Unilateral	Incomplete III pair	Corticosteroids Immunoglobulin	3 weeks

Ramakrishnan et al. 2015 [8]	30-year-old / female	Unilateral Photophobia Phonophobia	Incomplete III pair	Valproate Corticosteroids	1 month

Wang et al. 2014 [9]	63-year-old / male	Unilateral Pressure-like Phonophobia	Incomplete III pair	Valproate Flunarizine	NR

Shetty et al. 2013 [10]	26-year-old / female	Unilateral Throbbing	Incomplete III pair	NSAIDs Propranolol	2 weeks

NR: not reported; NSAIDs: nonsteroidal anti-inflammatory drugs.

**Table 3:** Mechanisms of action of drugs used in headache.

Drug	Mechanism of action
Corticosteroids	inhibit the synthesis of inflammatory proteins, induce the expression of anti-inflammatory proteins and inhibit cyclooxygenase-2 [11]

Indomethacin	inhibition of cyclooxygenase 1 and cyclooxygenase 2 and phospholipase A2 [12]

Valproate	increases gamma-aminobutyric acid (GABA) levels in synaptosomes and in the brain, reduces the central trigeminal nerve activation (by increasing GABA levels), was shown to reduce experimental neurogenic inflammation in the peripheral trigeminal vascular system [13]

Topiramate	modifies the excitability of nerves by blocking voltage-sensitive sodium channels and L-type voltage-activated calcium channels, inhibits carbonic anhydrase activity, inhibits the excitatory glutamate pathway while enhancing the inhibitory effect of GABA [13]

Pregabalin	binds to alpha 2-delta subunits of the voltage-gated calcium cannels in the nervous system, reducing the influx of calcium in the presynaptic ending, and, consequently, reduces the release of excitatory neurotransmitters including glutamate, noradrenaline, dopamine, and serotonin in the synaptic cleft [14]
